# Functional Properties of Rare Missense Variants of Human *CDH13* Found in Adult Attention Deficit/Hyperactivity Disorder (ADHD) Patients

**DOI:** 10.1371/journal.pone.0071445

**Published:** 2013-08-01

**Authors:** Thegna Mavroconstanti, Stefan Johansson, Ingeborg Winge, Per M. Knappskog, Jan Haavik

**Affiliations:** 1 K.G. Jebsen Centre for Research on Neuropsychiatric Disorders, University of Bergen, Bergen, Norway; 2 Division of Psychiatry, Haukeland University Hospital, Bergen, Norway; 3 Department of Biomedicine, University of Bergen, Bergen, Norway; 4 Department of Clinical Medicine, University of Bergen, Bergen, Norway; 5 Center for Medical Genetics and Molecular Medicine, Haukeland University Hospital, Bergen, Norway; University of Wuerzburg, Germany

## Abstract

The *CDH13* gene codes for T-cadherin, a GPI-anchored protein with cell adhesion properties that is highly expressed in the brain and cardiovascular system. Previous studies have suggested that *CDH13* may be a promising candidate gene for Attention Deficit/Hyperactivity Disorder (ADHD). The aims of this study were to identify, functionally characterize, and estimate the frequency of coding *CDH13* variants in adult ADHD patients and controls. We performed sequencing of the *CDH13* gene in 169 Norwegian adult ADHD patients and 63 controls and genotyping of the identified variants in 641 patients and 668 controls. Native and green fluorescent protein tagged wild type and variant CDH13 proteins were expressed and studied in CHO and HEK293 cells, respectively. Sequencing identified seven rare missense *CDH13* variants, one of which was novel. By genotyping, we found a cumulative frequency of these rare variants of 2.9% in controls and 3.2% in ADHD patients, implying that much larger samples are needed to obtain adequate power to study the genetic association between ADHD and rare CDH13 variants. Protein expression and localization studies in CHO cells and HEK293 cells showed that the wild type and mutant proteins were processed according to the canonical processing of GPI-anchored proteins. Although some of the mutations were predicted to severely affect protein secondary structure and stability, no significant differences were observed between the expression levels and distribution of the wild type and mutant proteins in either HEK293 or CHO cells. This is the first study where the frequency of coding *CDH13* variants in patients and controls is reported and also where the functional properties of these variants are examined. Further investigations are needed to conclude whether *CDH13* is involved in the pathogenesis of ADHD or other conditions.

## Introduction

During the past decade, major advances have been made in our understanding of the genetic underpinning of human disorders. It has increasingly been realized that many common illnesses are highly heritable and that a plethora of different genetic variants may contribute to the risk of complex somatic and psychiatric disorders. Furthermore, rare functional variants may be strongly associated with disease also in cases where no obvious association is observed for common genetic markers [1], [2]. For the common neurodevelopmental disorders, including schizophrenia, autism and ADHD, hypothesis-free linkage and genome wide association (GWA) studies have been particularly valuable, as these disorders generally lack good biological markers and there is a shortage of valid animal models that can provide insight into biological mechanisms [3]. As more susceptibility genes are discovered for psychiatric disorders, it has become clear that many of these genes are not only expressed in the brain and contribute to mental dysfunction, but are also involved in more general biological mechanisms and may contribute to a range of different disorders and phenotypes. This is illustrated in the search for ADHD susceptibility genes. To our knowledge, seven independent GWA studies in ADHD have been performed on different child and adult ADHD samples [4]–[11]. Although these studies did not identify any genes that were consistently associated with ADHD at a genome-wide level of significance, markers in certain genes were found to be strongly associated with ADHD in multiple studies. One of the most reproducible associations with ADHD was found for markers at the *CDH13* locus, that were ranked among the top results in four of the primary GWA studies and the meta-analyses of these studies, as well as a meta-analysis of five linkage scans [4], [12]. Furthermore, *CDH13* has been implicated in related psychiatric phenotypes such as methamphetamine and alcohol dependence, and depressive symptoms [13]–[15].

*CDH13* codes for T-cadherin (also known as truncated, H- or heart cadherin) that is an atypical member of the cadherin family of cell adhesion molecules. Unlike classical cadherins, it lacks a transmembrane domain, is attached to the cell membrane via a glycosylphosphatidylinositol (GPI) anchor and has low adhesive properties [16]–[19]. There is evidence that it functions as a negative guidance molecule during the development of the nervous system, and is involved in migratory processes, tumorigenesis and angiogenesis [20], [21]. In addition, CDH13 is involved in endoplasmic reticulum (ER) stress responses; in one study CDH13 was found to be upregulated in endothelial cells after induction of ER stress and to protect these cells from apoptosis by counteracting the proapoptotic response [22]. Another study identified GRP78, a molecular chaperone that participates, like CDH13, in pro-survival responses to ER stress_,_ as a signalling partner of CDH13 at the surface of endothelial cells [23].

T-cadherin is also a receptor for high molecular weight adiponectin and low-density lipoprotein and is highly expressed in the heart and cardiovascular system, skin and brain [18], [24], [25].

In several GWA studies *CDH13* has also been associated with serum adiponectin levels, metabolic syndrome, and cardiometabolic outcomes [26]–[29]. Most of these associations have reached genome-wide statistical significance [26], [27], [29]. Moreover, in a recent study in mice CDH13 was found to mediate the cardioprotective effects of adiponectin on cardiac stress [30]. Still, the functional roles of naturally occurring variants of this protein have not been investigated.

The aims of this study were to identify, functionally characterize, and estimate the frequency of coding *CDH13* variants in adult ADHD patients and controls.

## Materials and Methods

### Sequencing and Genotyping

To identify coding variants in *CDH13* we sequenced all 14 exons of the gene in 169 adult ADHD patients and a random sample of 63 adult controls using a standard dideoxy sequencing method. DNA was extracted from whole blood or saliva using the OrageneTM DNA Self-Collection Kit from DNA Genotek (DNA Genotek Inc., Ontario, USA). Primers were designed using Primer3, and the sequence analysis was performed on a 3730 DNA Analyzer (Applied Biosystems). All sequences were manually inspected using the SeqScape software (Applied Biosystems). The variants that were identified in the sequencing study were genotyped in a larger Norwegian sample of adult ADHD patients (n = 641) and controls (n = 668) using the MassARRAY iPLEX System (Sequenom, San Diego, CA). Protocols for PCR amplifications and fragment analysis are available upon request. The final genotyping call rate was >0.99.

### Analysis of Genotyping Results

PLINK was used for the calculation of allele frequencies in the patient and control samples [31], online information at: http://pngu.mgh.harvard.edu/purcell/plink/. Two-tailed P-values for genotype frequencies were calculated by Fisher’s exact test, in a 2×2 contingency table, using the free Graphpad QuickCalcs software that is available online at http://www.graphpad.com/quickcalcs/.

### Subjects and Measures

Patients were mainly recruited from a national ADHD registry, but also by psychiatrists and psychologists working in outpatient clinics [32]. A clinical diagnosis of ADHD/hyperkinetic disorder was made according to ICD-10 or DSM-IV criteria [33]. Controls were randomly recruited from the Norwegian population through the Medical Birth Registry of Norway [32]. There were no exclusion criteria for the controls.

### Ethics Statement

All participants signed the written informed consent form, and the study was approved by the Norwegian Regional Medical Research Ethics Committee West (IRB #3 FWA00009490, IRB00001872).

### *In silico* Analysis

*In silico* analysis of the effect of *CDH13* variants identified in our sample was performed using PolyPhen v.2.2.2 and Sorting Intolerant From Tolerant (SIFT), online tools available at http://genetics.bwh.harvard.edu/pph2/and http://sift.jcvi.org/, respectively. I-mutant 3.0, an online Support Vector Machine tool based on ProTherm [34] that is available at http://gpcr.biocomp.unibo.it/cgi/predictors/I-Mutant3.0/I-Mutant3.0.cgi was used in addition to predict protein stability changes. The three-dimensional structure of full-length CDH13 has not been experimentally determined. The NMR structure of the N-terminal domain of human CDH13 was reported in 2008 [19] whereas the crystal structure of the first two domains of mouse and chicken CDH13 and the first domain in Xenopus Laevis was recently published [35]. Our *in silico* analyses were therefore based on the human CDH13 protein sequence P_001248.1. The SIFT prediction, as previously described in [36], [37], is based on an analysis of amino acid conservation in an alignment of related sequences and reports a tolerance score for a specific amino acid substitution. Variants with scores below 0.05 were considered damaging and variants with scores above 0.05 were considered as being tolerated. We performed a SIFT prediction of human SNPs that was based on GRCh37, ensemble 55. The PolyPhen prediction, as previously described in [38], is based on sequence, phylogenetic and structural feature analysis and reports a score for each amino acid substitution which is classified as benign, possibly damaging and probably damaging. I-mutant 3.0, as previously described in [39], [40], is a tool used for the automatic prediction of the effects of single point mutations on protein stability by calculating the unfolding Gibbs free energy value of the mutant proteins minus that of the wild type (wt) protein (ΔΔG = ΔG mutant – ΔG wild type), given in kcal/mol. For this analysis, the predictor of protein stability changes upon single point mutation was selected and the ternary classification system of stability prediction (SVM3) according to which, −0.5< = ΔΔG< = 0.5 corresponds to neutral stability, ΔΔG<−0.5 to a large decrease of stability and ΔΔG >0.5 to a large increase of stability. The conditions were set at pH 7.0 and 25°C, and a reliability index (RI: 0 −10) was given for large stability decreases or neutral stability.

### Expression Vectors and Sequences

**Figure 1:**
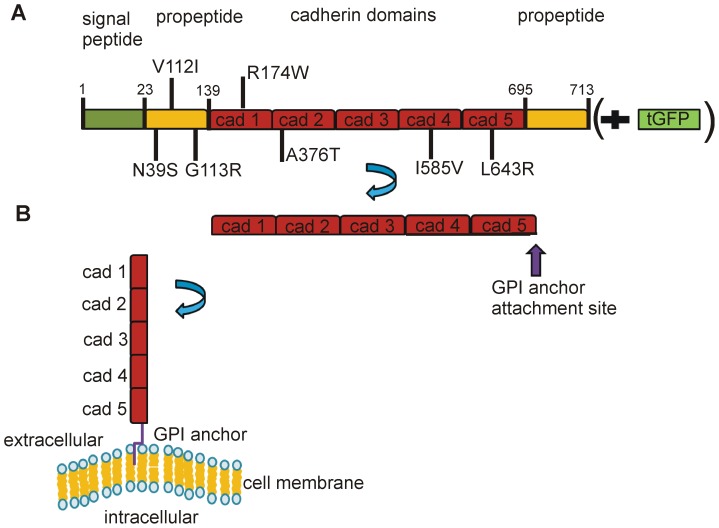
Schematic description of the CDH13 proteins expressed in CHO and HEK293 cells. CDH13 was expressed with or without a C-terminal tGFP tag in HEK293 and CHO cells, respectively. The location of the identified variants is also shown (A). According to the general model of processing of GPI anchored proteins in the ER, a C-terminal transmembrane domain is cleaved off and is then replaced by a GPI anchor. The protein with the attached GPI anchor is then directed to the external side of the plasma membrane (B). Wild type and variant CDH13 proteins were expressed on the cell membrane in HEK293 and CHO cells. In HEK293 cells, the C-terminal GFP tag of the GFP-CDH13 fusion proteins was cleaved off as a result of GPI anchoring at the c- terminal of CDH13 and the fully processed protein was subsequently transferred to the cell membrane.

For expression studies in Chinese hamster ovary (CHO) and human embryonic kidney cells (HEK293) cells, two eukaryotic expression vectors were used carrying the wild type *CDH13* sequence (clone BC030653) : 1) pcmv_6_AC_wt CDH13_GFP, (RG206068, Origene Technologies, Rockville, USA) and 2) pCI-neo (the empty vector was kindly provided by Hanne Ravneberg, UniTargetingResearch AS, University of Bergen). To obtain pCI-neo_wt CDH13 the cDNA clone BC030653 was subcloned from pBluescript (Thermo scientific) into pCI-neo at the MluI and NotI restriction sites. The wild type *CDH13* sequence corresponding to NM_001257.4, and the identified variants were obtained by mutagenesis according to the manufacturer’s instructions (Stratagene, QuikChange™ site- directed mutagenesis kit). Primer sequences are available upon request. A schematic representation of the proteins expressed in CHO and HEK293 cells is shown in Fig. 1.

### Cell Culture and Transfections

HEK293 cells, as described previously [41], and CHO cells (Sigma Aldrich) were grown as adherent monolayers in a 5% CO_2_ humidified atmosphere, at 37°C, in DMEM-F12, without phenol red (Invitrogen), supplemented with 10% foetal bovine serum (SAFC, Sigma-Aldrich) and 1 ml gentamicin (Sigma-Aldrich). CHO cells were transiently transfected with pCI-neo_wild type or mutant CDH13 using lipofectamine LTX & plus reagent (Invitrogen) according to the manufacturer’s instructions. HEK293 cells were transiently transfected with pcmv_6_AC_GFP, carrying GFP-tagged wild type or mutant *CDH13*, using Magnet Assisted Transfection according to the manufacturer’s instructions (MATra, IBGmbH, Göttingen, Germany). Mock transfected cells were transfected with the corresponding empty vectors.

### Imaging

Imaging of living HEK293 cells expressing the GFP-CDH13 fusion proteins was performed on a NIKON TE2000 (Nikon, Tokyo, Japan) fluorescence microscope using a 40×objective. Imaging of fixed and stained cells was performed on a Leica TCS SP5 confocal microscope (Leica microsystems, Wetzlar, Germany) using a 63×objective and 5×zoom in. Imaging was performed at the Molecular Imaging Center (Fuge, Norwegian Research Council), University of Bergen.

### Gel Electrophoresis and Immunoblot

Total cell lysate from HEK293 and CHO cells was obtained at 48 and 24 hours post-transfection, respectively, using Radio-Immunoprecipitation Assay (RIPA) lysis buffer (Sigma-Aldrich) supplemented with a protease inhibitor cocktail (Roche). The lysate was clarified by centrifugation for 10 minutes at 10,000×g. The supernatant was used for protein quantification by a Bradford assay (Bio-Rad) and 40 µg protein/sample was loaded on a 4–15% SDS-polyacrylamide gel (Biorad). Proteins were separated by electrophoresis and subsequently transferred onto a nitrocellulose membrane (Whatman International Ltd, UK). The membrane was blocked in 5% non-fat dry milk (Biorad) for one hour followed by overnight incubation with the primary antibody (for details see section below: Antibodies) at 4°C. The next day the membrane was incubated with the secondary antibody for one hour at room temperature. Pierce ECL western blotting substrate was applied before chemiluminescent imaging (Thermo scientific).

### Immunocytochemistry

Transiently transfected cells were grown overnight on sterile poly-lysine-coated (Sigma-Aldrich) coverslips. At 24 h (CHO) or 48 h (HEK293) post tranfection, the cells were fixed in 4% paraformaldehyde for 10 min (Polysciences Europe GmbH) and blocked in 1% Bovine serum albumin (Sigma-Aldrich) for 1 h. The cells were subsequently incubated with the primary and secondary antibodies for 1 h each at room temperature. At the end of antibody incubation, the cells were washed and the coverslips were mounted on glass slides with mounting medium containing DAPI (ProLong® Gold, Invitrogen). Alternatively, after fixation, the cells were kept in PBS at 4°C for up to four weeks before proceeding to blocking and staining. For CDH13 stainings in CHO cells, the cells were permeabilized for 10 min in a solution containing 0, 1% Triton-X 100 (Sigma-Aldrich) before blocking.

### Antibodies

Immunoblotting was performed using the following primary antibodies: a goat polyclonal antibody against CDH13, immunogen: Glu23-Ala692,(AF3264), from R&D Systems (5∶1000), a mouse monoclonal against t-GFP (2H8, TA150041) from Origene Technologies (1∶700) and a mouse monoclonal against a-tubulin (T9026) from Sigma-Aldrich (2∶1000). The following HRP-conjugated secondary antibodies were used: a donkey anti-goat, ab6885-1, from Abcam (1∶5000) and a goat anti-mouse (170–6516), from Biorad (1∶3000).

For immunocytochemistry the following antibodies were used: a goat polyclonal antibody against CDH13 (AF3264) from R&D Systems (1∶40), an anti-goat secondary antibody conjugated to NL557 (NL001) from R&D Systems (1∶400).

## Results

### Sequencing and Genotyping

The results of the sequencing and genotyping studies of *CDH13* are summarized in Table 1. Sequencing revealed seven coding variants in the total sample (n = 232) of ADHD patients (n = 169) and controls (n = 63). Of these variants, only R174W was novel. All seven variants were identified in the patients (accumulated allele frequency 4.6%) whereas only four of these were found in the controls (accumulated allele frequency 3.9%). Targeted genotyping in a larger population sample (n = 1309) detected all seven variants in both the patient (n = 641) and control (n = 668) groups with an accumulated allele frequency of 3.2% in patients and 2.9% in controls. None of the *CDH13* variants showed a significant association with ADHD either individually or in combination.

**Table 1:** Frequency of *CDH13* variant alleles identified in Norwegian adult patient and control groups.

Variant		Allele Frequencies Sequencing			Allele Frequencies Genotyping		
		169 Cases	63 Controls	P value	641 Cases	668 Controls	P value
V112I	rs200199969	0.88%	0.79%	1.00	0.46%	0.74%	0.45
G113R	rs183971768	0.29%	0.79%	0.47	0.23%	0.37%	0.49
R174W	novel	0.29%	0%	1.00	0.07%	0.14%	1
A376T	rs35549391	1.15%	0%	0.57	0.78%	0.59%	0.63
I585V	rs199759196	0.55%	0%	1.00	0.23%	0.07%	0.36
L643R	rs34106627	0.55%	1.55%	0.29	0.15%	0.14%	1
N39S	rs72807847	0.88%	0.79%	1.00	1.32%	0.82%	0.18
Total		4.59%	3.9%	0.80	3.24%	2.87%	0.64

Seven *CDH13* variants were identified in the sequencing study, three of which were only detected in patients. All variants were genotyped in a larger sample. Two-tailed P-values for genotype frequencies were calculated by Fisher’s exact test in a 2×2 contingency table.

### *In silico* Prediction of the Effects of the Identified CDH13 Variants

The results of the *in silico* predictions of the effects of the CDH13 variants identified in our sample are shown in Table 2. Both SIFT and Polyphen predicted the R174W mutation to be damaging for the protein, whereas Polyphen also predicted the G113R mutation to be probably damaging. The rest of the variants were predicted to be tolerated or benign. Furthermore, according to the I-mutant 3.0 prediction, all the variants were estimated to have a lower stability compared to the wild type protein. According to the ternary classification (SVM3), however, only the I585V and L643R have a ΔΔG<−0.5, which corresponds to a large decrease of stability.

**Table 2:** *In silico* analysis of the effect of CDH13 variants.

Variant		SIFT (score)	Polyphen (score)	ΔΔG (RI)
V112I	rs200199969	Tolerated (0.32)	Benign (0.004)	−0.45 (4)
G113R	rs183971768	Tolerated (0.23)	Probably damaging (0.993)	−0.40 (2)*
R174W	novel	Damaging (0.01)	Probably damaging (1)	−0.45 (3)
A376T	rs35549391	Tolerated (0.29)	Benign (0.270)	−0.59 (6)
I585V	rs199759196	Tolerated (1)	Benign (0.003)	−1.17 (7)
L643R	rs34106627	Tolerated (0.22)	Benign (0.270)	−1.43 (3)
N39S	rs72807847	Tolerated (0.44)	Benign (0)	−0.11 (1)

The analysis was based on the protein sequence. SIFT scores below 0.05 were considered damaging. I-mutant-3.0 predicted the effects of the variants on protein stability by calculating the unfolding Gibbs free energy value of the mutant proteins minus that of the wild type protein (ΔΔG = ΔG mutant – ΔG wild type), given in kcal/mol. A negative change indicates decreased stability. The reliability index (RI) for a large decrease (ΔΔG<−0.5) ranged from 0–10. For the G113R* a reliability index for a neutral stability change was given. Ternary classification (SVM3) −0.5< = ΔΔG< = 0.5 corresponds to neutral stability, ΔΔG<−0.5 to large decrease of stability and ΔΔG >0.5 to a large increase of stability.

### Expression Levels of Wild Type and Mutant CDH13 Protein

**Figure 2:**
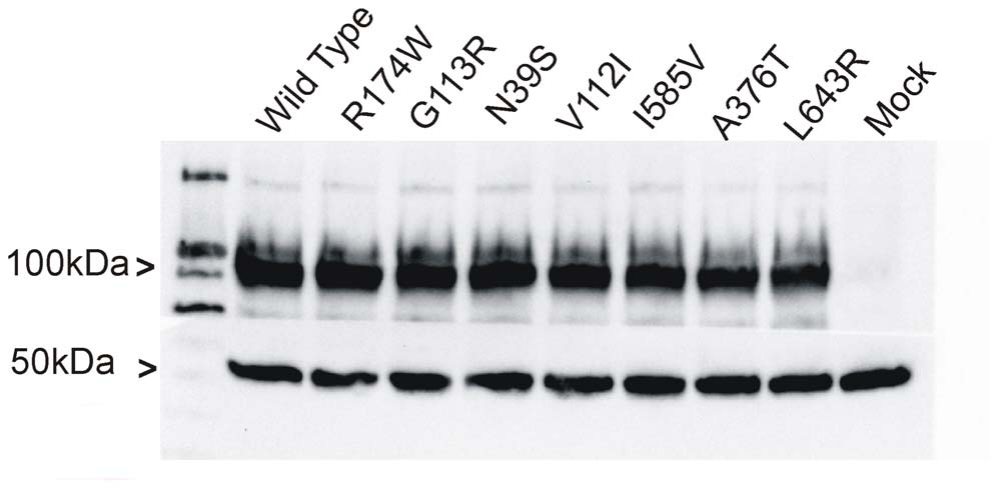
Expression levels of wild type and variant CDH13 proteins in CHO cells. Western blot results: In A) wild type and variant CDH13 proteins (105 kDa) were detected in CHO cells by an antibody against CDH13 (AF3264). Mock cells transfected with the empty vector did not express CDH13. A-tubulin (50 kDa), the protein loading control,was detected by an antibody against a-tubulin (T9026).

Wild type CDH13, as well as the seven coding variants of CDH13 that had been detected in patients or controls, were transiently expressed in CHO cells. Using western blotting with an antibody against CDH13, a major protein band of approximately 105 kDa, which was absent in mock transfected cells, was detected in CHO cells expressing CDH13 proteins (Fig. 2).

To further investigate the morphology and processing of CDH13 protein in living cells, C-terminally GFP-tagged wild type and variant fusion proteins were expressed in HEK293 cells. In these cells a major band of approximately 131 kDa was detected using an antibody against GFP (Fig. S1A). However, two bands were detected when an antibody against CDH13 was used, one at approximately 131 kDa and another at 105 kDa (Fig. S1B). In control HEK293 cells that were transfected with the empty GFP vector, only a single band at approximately 26 kDa was detected, corresponding to GFP (Fig. S1A). Neither the CDH13-GFP fusion proteins nor the native CDH13 protein were present in control HEK293 cells (Fig. S1A and S1B). Expression levels and molecular weights of the wild type protein and the variants were comparable and no obvious differences were observed in at least three independent western blot experiments with each variant (Fig. 2 and Fig. S1A and S1B).

### Immunocytochemistry

**Figure 3:**
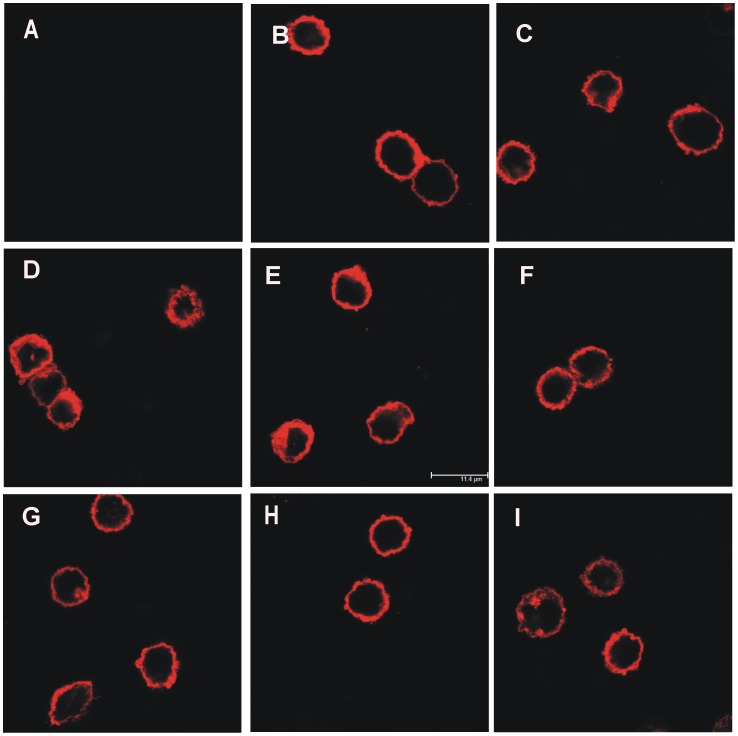
CDH13 stained CHO cells expressing wild type and variant CDH13 on the plasma membrane. Cells were permeabilised before staining. A) Mock transfected cells, B) wild type CDH13, C) A376T, D) G113R, E) I585V, F) L643R, G) N39S, H) R174W, I) V112I. Wild type and variant CDH13 proteins were expressed on the cell membrane. Mock transfected cells did not express CDH13.

Immunostainings using an antibody against CDH13 were performed in permeabilized CHO cells and the cellular localization of wild type and variant CDH13 proteins was examined by confocal microscopy. Confocal images showed only plasma membrane localization of wild type and variant CDH13, which was absent in mock transfected cells (Fig. 3). To study the subcellular localization of the CDH13-GFP fusion proteins in HEK293 cells, we obtained fluorescence wide field images of living cells, and confocal images of fixed cells immunostained for membrane bound CDH13. GFP staining was not necessary since the GFP fluorescent signal could still be detected in fixed and stained cells. In living HEK293 cells, localization of wild type and variant GFP-CDH13 proteins was observed in the cytoplasm and was different from the uniform intracellular localization of GFP that was observed in mock cells (Fig. S2). The same GFP signal (green) was observed in the cytoplasm of cells stained for membrane bound CDH13 (red) (Fig. S3). Cell surface CDH13 was detected by the same antibody that detected the 105 kDa protein band in Fig. 2 and S1B. Cell surface expression of CDH13 was absent in mock transfected cells (HEK293-GFP) (Fig. S3).

## Discussion

### CDH13 as an ADHD Susceptibility Gene

As ADHD is a clinically and probably also etiologically complex disorder, it is not surprising that it has been difficult to identify susceptibility genes of strong effect [3]. Still, the *CDH13* gene has been implicated in ADHD and related phenotypes in several large genetic studies and meta-analyses [4], [5], [12], [42], [43]. Moreover, the pattern of brain expression of CDH13 and its presumed role in migratory processes of the developing brain, makes this a strong candidate for neurodevelopmental disorders [4]. It has been suggested that CDH13 is not only an adhesion molecule and a receptor for extracellular ligands, but that it can also trigger intracellular signaling systems by interaction with other membrane bound molecules, including transmitter receptors located in lipid rafts [4], [23].

To our knowledge, this is the first investigation of *CDH13* coding variants in a large sample of patients and controls. DNA sequencing revealed seven *CDH13* missense variants, one of which was novel and three were only found in patients. Genotyping of these variants in 641 ADHD patients and 668 controls, however, did not reveal significant association with ADHD. This is probably due to limited statistical power, as the allele frequency of the variants was overall low in both samples. The N39S missense variant was the only variant with allele frequency above 1%, (1.3%) in our patient sample. Out of the seven variants we identified, only G113R has been previously reported in connection with a phenotype. CDH13-G113R was one out of five CDH13 mutations identified in amyotrophic lateral sclerosis (ALS) patients, all of which were absent in controls [44].There was, however, no evidence of any effects of *CDH13* variants in ALS in that study.

Based on the observed frequency of mutations in patients and controls (Table 1), we performed power calculations to estimate the number of samples required to obtain statistical significance. Assuming a combined frequency of rare coding variants of 3%, and an expected odds ratio of 1.5 per risk allele, a sample of 1250 cases (1∶1 ratio of cases to controls) would be needed to obtain 80% power at the p = 0.05 level, and 4900 samples at the 2.5×10^−6^ level (controlling for testing of 20 000 genes). To test a single variant of 1% minor allele frequency, the corresponding sample sizes would be 3600 and 14000, respectively (at P = 0.05 and P = 2.5×10^−6^). Thus, much larger samples than available in the current study are needed to obtain statistical power to address the role of rare CDH13 mutations in ADHD, or other complex phenotypes. In addition, novel methods and statistical approaches might be needed to identify risk genes and their role in such disorders. In a recent study a new multivariate approach was used to identify genes with significant association to neuroimaging measures of brain function in a sample of elderly Alzheimer’s disease patients and elderly people with mild cognitive impairment [45]. *CDH13* was one of 22 genes that were significantly associated with temporal lobe volume [45].

### *In silico* Prediction of the Effects of CDH13 Missense Variants on Protein Function

Missense variants that are associated with Mendelian disorders typically interfere with protein stability, folding, solubility or cellular processing [46]. Many of these effects can be predicted using *in silico* analyses (Table 2). The SIFT and Polyphen results were mostly consistent, showing that most of the variants were tolerated or benign. Likewise, the R174W variant was predicted by both SIFT and Polyphen to be damaging or probably damaging. The G113R variant, however, was only predicted by Polyphen to be probably damaging. This is most likely due to differences in analysis parameters used in SIFT [36], [37] and Polyphen 2 [38]. The I-mutant-3 results showed decreased stability for all the variants. Larger stability decreases, however, were predicted only for the I585V and L653R variants. Discrepancies in the prediction results obtained from different *in silico* analysis tools are expected as shown in several similar studies [47], [48]. These are probably due to the overall limitations of *in silico* analysis and the limitations encountered, for instance, due to lack of structural or functional data for many proteins.

As predicted from their position in the CDH13 molecule, the missense variants studied here might be involved in several aspects of CDH13 function. The N39S, V121I and G113R mutations are all located in the pro-peptide domain, where they could interfere with proper folding and processing of the immature CDH13 protein. The R174W mutation is localized in the middle of the extracellular domain 1 (EC1) of the mature protein, in a beta strand structure that is highly conserved in mammalian CDH13. It is also close to the residues involved in the recently identified ”X-dimer” dimerization configuration between EC1 and EC2 of CDH13, which is important for homodimerization, adhesion and neurite outgrowth [35]. Thus, R174W might influence these functions of CDH13, as well as its angiogenic effects that are mediated by EC1 and EC5 [49].

CDH13 is a glycoprotein containing several glycosylation sites where N-linked oligosaccharides are attached and promote proper folding in the ER and protein stability [50]. The N39S, A376T, I585V and L643R variants are in close proximity (6–13 amino acid residues) to N glycosylation sites. However, none of the variants are located at a glycosylation site so it is unlikely that any of them cause misfolding or instability by preventing proper glycosylation. This is also demonstrated by the molecular weight which is the same for all the CDH13 variants studied here.

The missense variants A376T, I585V and L643R are found in extracellular domains 2, 4, and 5, respectively. The L643R is located about fifty amino acid residues from the GPI anchor in the mature protein [50]. The functional roles of these residues and protein domains are less clear and since the *in silico* modeling predicted the mutations to be benign, the lack of clear effects of the missense variants was not unexpected.

### CDH13 Protein Expression, Intracellular Localization and Function of CDH13

In previous studies on human aortic smooth muscle cells CDH13 has been identified as a cell surface expressed, LDL-binding protein of around 130 kDa and 105 kDa [51]. The cell surface expression pattern and LDL-binding properties were also shown in HEK293 cells transfected with CDH13 [52]. Immunostaining of endogenously expressed CDH13 in a human keratinocyte cell line (DJM-1) revealed a band of approximately 105 kDa [18]. In most studies CDH13 has been found at the extracellular surface of the plasma membrane [52], [53]. However, expression in other cellular compartments, such as the nucleus and centrosomes in endothelial cells, has also been reported [54]. Expression of CDH13 was also observed in neural cytoplasm as well as membrane and neurites in staining of the adult human cerebral cortex [25].

Our findings show that native wild type and variant CDH13 proteins, of approximately 105 kDa (Fig. 2), are expressed in CHO cells on the cell membrane (Fig. 3). In line with previous findings [16], [18] we did not observe increased signal between adjacent cells or CDH13 accumulation at sites of intercellular contacts in CDH13 stained cells, as is commonly observed with classical cadherins such as E-, N-[55] and P-cadherin [18]. The wild type protein and the seven variants showed similar expression levels and localization. In order to detect partial intracellular accumulation of abnormal CDH13, which would be missed by membrane staining, we permeabilised the cells before staining for CDH13. Cytoplasmic localization of membrane proteins may represent abnormal accumulation in the ER which is commonly associated with disease. For instance, in the case of Crohn’s disease-associated SNPs in E-cadherin, a variant was associated with the formation of a truncated E-cadherin, and cytoplasmic accumulation, instead of membrane expression, in the intestinal epithelium of affected patients and in transfected cells [56]. Our findings in CHO cells, however, show no such effects of the CDH13 variants studied here.

We also observed similar expression levels and localization of wild type and variant CDH13 in living HEK293 cells expressing GFP-CDH13 fusion proteins (see supplementary information). T-cadherin is a GPI anchored plasma membrane protein. Canonical processing of GPI-anchored proteins involves C-terminal cleavage and attachment of a GPI anchor in the endoplasmic reticulum (ER), followed by Golgi–mediated plasma membrane transport and localization [57]. This is schematically presented in Fig. 1. The wild type and all the variant GFP-CDH13 proteins showed comparable expression levels as GFP-CDH13 fusion proteins of approximately 131 kDa, and as C-terminally processed GFP-lacking CDH13 proteins of approximately 105 kDa (Fig. S1A and S1B). All the fusion proteins (green) also showed similar distribution in the cytoplasm in unstained living cells (Fig. S2), whereas the C-terminally processed CDH13 (red) showed plasma membrane distribution in fixed and CDH13 stained cells (Fig. S3). Although GFP tags have been used successfully to study N-cadherin [58] and E-cadherin [59] function there is also the possibility that this tag might interfere with the normal processing and functions of the protein being studied. Thus, in addition to plasma membrane localized CDH13 (red signal) (Fig. S3) we also observed a strong green signal from all the wild type and mutant CDH13-GFP fusion proteins (Fig. S2 and S3) in the cytoplasm. However, we consider that this signal, which was not observed in CHO cells expressing the native proteins, is a GFP-related artefact and is therefore not biologically relevant. This illustrates that GFP-tags should be used with caution. Despite this limitation, the GFP-fusion expression model facilitated the observation of canonical GPI anchor processing of CDH13 with the concomitant expression of CDH13 lacking the GFP tag on the cell membrane. In summary, the cytoplasmic signal from the fusion proteins which can be considered a related GFP artefact does not affect our conclusions that, as the native proteins in CHO cells, the wild type and variant CDH13- GFP fusion proteins were equally expressed, processed and localized on the cell membrane.

### Conclusions

In this study we tested the association of *CDH13* with adult ADHD by sequencing the *CDH13* gene in a Norwegian sample of ADHD patients and controls. This was followed by genotyping the identified *CDH13* variants in a larger sample.

However, assuming a moderate effect size, this study is probably underpowered to detect significant associations between rare CDH13 variants and ADHD. To investigate the functional effects of CDH13 variants we expressed the wild type protein and the missense variants as native CDH13 in CHO cells and as GFP fusion proteins in HEK293 cells. In both models, we could observe the canonical processing of CDH13 as a GPI anchored protein. In the HEK293 cell lines a C-terminal sequence, which also includes a GFP tag, is cleaved off and replaced by a GPI anchor. We obtained similar results from the two over-expression models we used showing comparable levels of protein expression and cell membrane localization of wild type and variant CDH13 proteins. This however, does not exclude the possibility that these CDH13 variants may affect some functions of CDH13 that have not been examined in the current study.

## Supporting Information

Figure S1 **Expression levels of wild type and variant GFP-CDH13 fusion proteins in HEK293 cells.** Western blot results: In A), GFP-CDH13 fusion proteins (26 kDa+105 kDa = 131 kDa) were detected in HEK293 cells by an antibody against GFP (TA150041). Mock cells transfected with the empty GFP vector expressed only GFP (26 kDa). In B), GFP-CDH13 fusion proteins were detected by an antibody against CDH13 (AF3264). Two bands were detected by this antibody, one at approximately 131 kDa and another at 105 kDa. Mock cells transfected with the empty GFP vector did not express CDH13. In A), B) the protein loading control, A-tubulin (50 kDa), was detected by an antibody against a-tubulin (T9026).
(TIF)

Figure S2 **Localization of GFP-CDH13 fusion proteins in living HEK293 cells.** Images of living cells showed cytoplasmic localization of GFP-CDH13. In mock cells GFP was distributed all over the intracellular space. A) HEK293-GFP, B) HEK293-Wild Type CDH13, C) HEK293-GFP-A376T, D) HEK293-GFP-G113R, E) HEK293-GFP-I585V, F) HEK293-GFP-L643R, G) HEK293-GFP-N39S, H) HEK293-GFP-R174W, I) HEK293-GFP-V112I.
(TIF)

Figure S3 **CDH13 stained HEK293 cells expressing GFP-wild type and variant CDH13 fusion proteins.** Two distinct signals were observed in cells stained for cell surface CDH13∶1. GFP-CDH13 (green) localized in the cytoplasm as it was observed in living cells and 2. CDH13 expressed on the cell membrane (red). Mock cells transfected with GFP did not express CDH13. A) HEK293-GFP, B) HEK293-Wild Type CDH13, C) HEK293-GFP-A376T, D) HEK293-GFP-G113R, E) HEK293-GFP-I585V, F) HEK293-GFP-L643R, G) HEK293-GFP-N39S, H) HEK293-GFP-R174W, I) HEK293-GFP-V112I.
(TIF)
