# Supplementary figures and images for: Functional Properties of Rare Missense Variants of Human CDH13 Found in Adult Attention Deficit/Hyperactivity Disorder (ADHD) Patients
Source: PLoS One. 2013 Aug 1;8(8):e71445. doi: 10.1371/journal.pone.0071445 (PMC3731280; doi:10.1371/journal.pone.0071445)

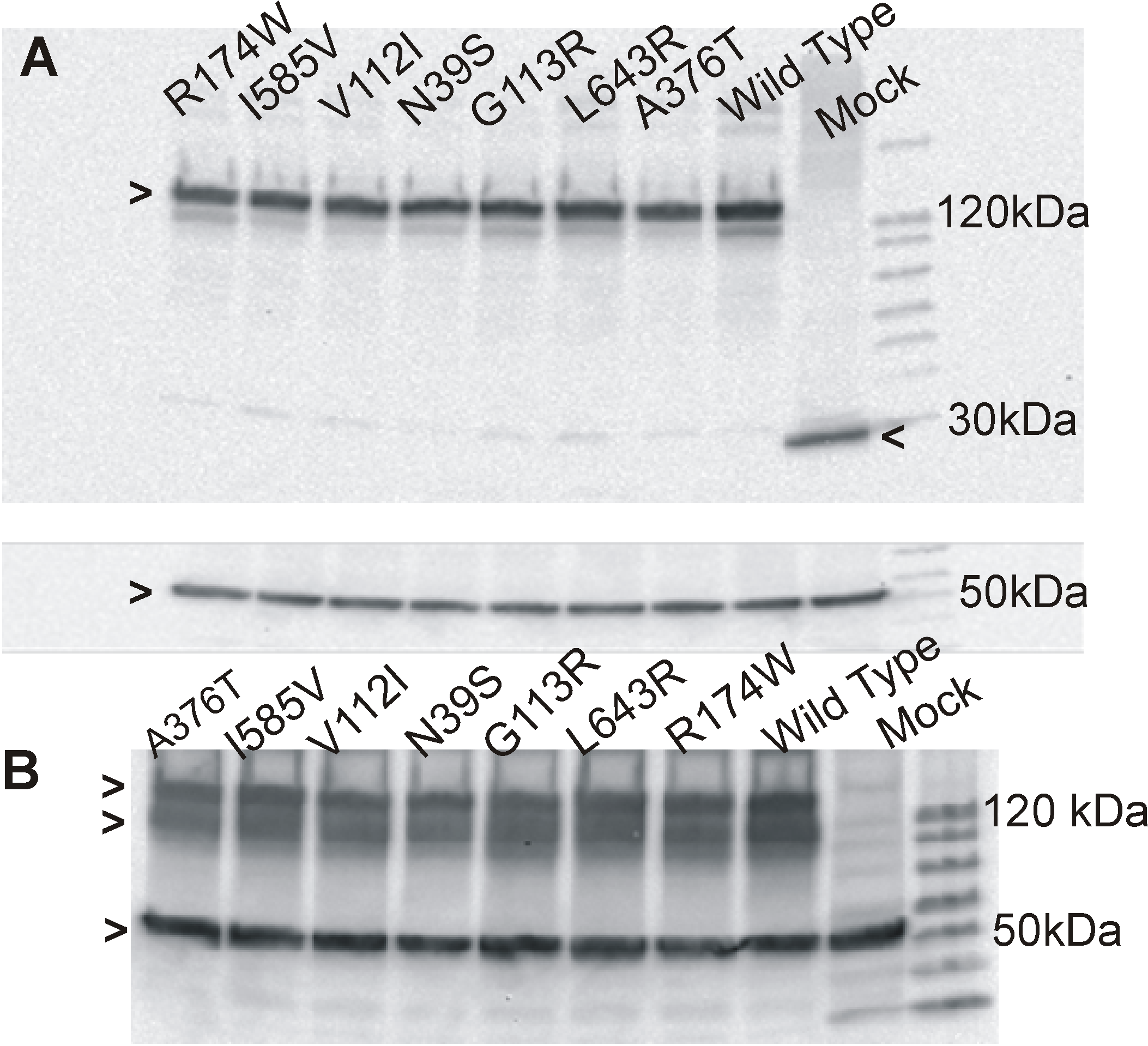

Supplement: Figure S1 — Expression levels of wild type and variant GFP-CDH13 fusion proteins in HEK293 cells. Western blot results: In A), GFP-CDH13 fusion proteins (26 kDa+105 kDa = 131 kDa) were detected in HEK293 cells by an antibody against GFP (TA150041). Mock cells transfected with the empty GFP vector expressed only GFP (26 kDa). In B), GFP-CDH13 fusion proteins were detected by an antibody against CDH13 (AF3264). Two bands were detected by this antibody, one at approximately 131 kDa and another at 105 kDa. Mock cells transfected with the empty GFP vector did not express CDH13. In A), B) the protein loading control, A-tubulin (50 kDa), was detected by an antibody against a-tubulin (T9026). (TIF) [file pone.0071445.s001.tif]

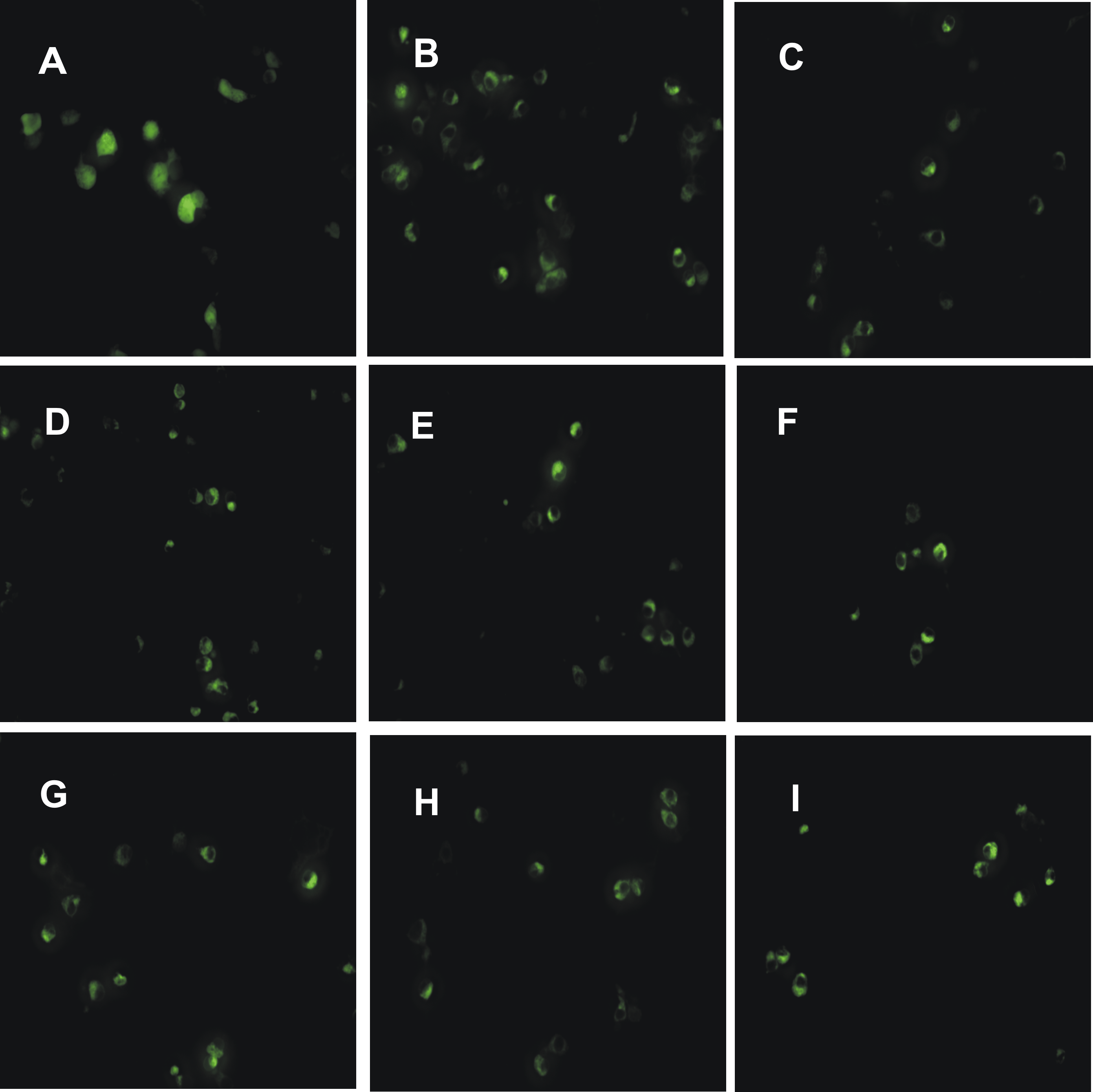

Supplement: Figure S2 — Localization of GFP-CDH13 fusion proteins in living HEK293 cells. Images of living cells showed cytoplasmic localization of GFP-CDH13. In mock cells GFP was distributed all over the intracellular space. A) HEK293-GFP, B) HEK293-Wild Type CDH13, C) HEK293-GFP-A376T, D) HEK293-GFP-G113R, E) HEK293-GFP-I585V, F) HEK293-GFP-L643R, G) HEK293-GFP-N39S, H) HEK293-GFP-R174W, I) HEK293-GFP-V112I. (TIF) [file pone.0071445.s002.tif]

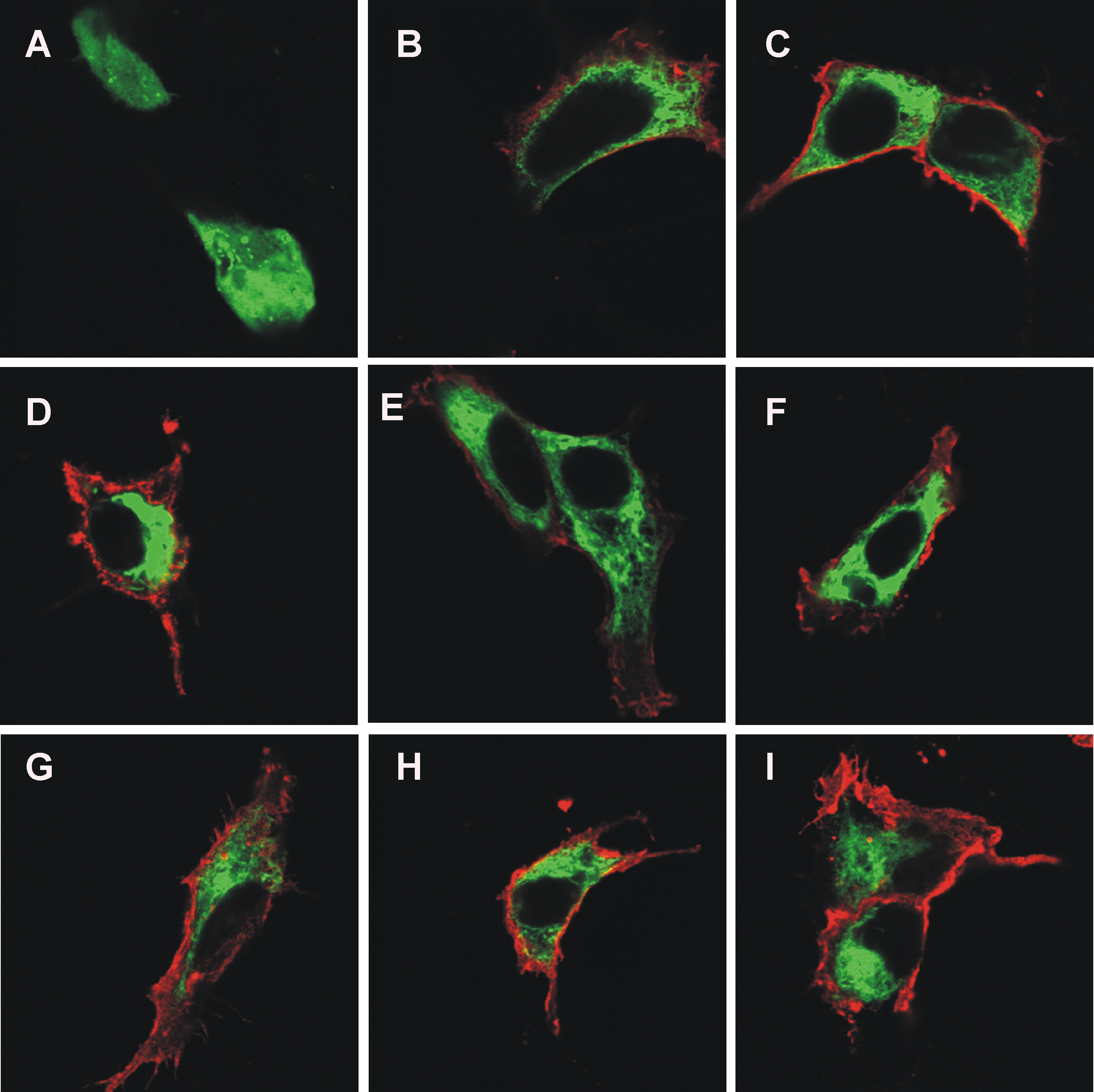

Supplement: Figure S3 — CDH13 stained HEK293 cells expressing GFP-wild type and variant CDH13 fusion proteins. Two distinct signals were observed in cells stained for cell surface CDH13∶1. GFP-CDH13 (green) localized in the cytoplasm as it was observed in living cells and 2. CDH13 expressed on the cell membrane (red). Mock cells transfected with GFP did not express CDH13. A) HEK293-GFP, B) HEK293-Wild Type CDH13, C) HEK293-GFP-A376T, D) HEK293-GFP-G113R, E) HEK293-GFP-I585V, F) HEK293-GFP-L643R, G) HEK293-GFP-N39S, H) HEK293-GFP-R174W, I) HEK293-GFP-V112I. (TIF) [file pone.0071445.s003.tif]
